# Specific Treatment of Necrosis of the Alveoli and Maxillæ with Aromatic Sulphuric Acid

**Published:** 1896-05

**Authors:** W. A. Mills

**Affiliations:** Baltimore, Md.


ARTICLE VII.

### SPECIFIC TREATMENT OF NECROSIS OF THE ALVEOLI AND MAXILLÆ WITH AROMATIC SULPHURIC ACID.

BY W. A. MILLS, D. D. S., BALTIMORE, MD.

Read in the Section on Dental and Oral Surgery, at the Forty-Sixth Annual Meeting of the American Medical Association, at Baltimore, Md., May 7-10, 1895.

I desire to bring before your attention the non-operative treatment of necrosis of the alveoli and maxillæ with aromatic sulphuric acid. I shall cite only two cases in office practice, entering as little as possible into any minute detail of their history or etiology.

Case 1.—A lady of nervo-bilious temperament, aged 30, called to consult me about a fistulous opening situated at the right side of the superior central incisor which was discharging freely a dark-colored pus. On examination, I found the superior right central and lateral incisors and cuspid teeth dead. Patient informed me they had been so for seven years or more, and had never been treated. I diagnosed the case as necrosis superinduced by chronic abscesses. I prescribed the following:

R.—Acid sulphuric aromatic, - ℥ ij. 60
Aqua, - - f℥ x. 300
Misce.

To be injected by the patient into the fistulous opening five or six times daily.* The patient was also instructed to visit the office every other day for examination.

At the expiration of two weeks all discharge had ceased, the soft tissues had fallen into the cavity, made by the chemic action of the aromatic sulphuric acid upon the necrosed tissue. An incision was made extending from the fistulous opening to the right first bicuspid. The cavity was then packed with absorbent cotton saturated with the following:

R.—Acid carbolic

Tinct. iodin, - aa ʒ ss 2

Aqua, - - f℥ xij 360

Misce.

Patient was then dismissed to return the next day, when cotton pledget was removed. The soft tissues having been pushed aside, I was able to see to what extent the bony structures had been diseased. I found the line of necrosis had involved the right facial surface of the superior maxilla from the left central to the right bicuspid teeth and upward from the alveolar ridge to the anterior nasal spine, a part of which with bony structure around the apices of the dead teeth had been destroyed; only sufficient alveolar sputum remained to hold them in position.† I opened the nerve canals of the dead teeth and

*Bicarbonate of soda and water, used as alkalin mouth wash after every injection.

†A similar case is noted by Dr. Black in American System of Dentistry, vol. 1, p. 950.

cleaned, disinfected and filled them at once. The filling material was forced through the apical foramina, from the posterior surface, and dressed in the usual manner. The patient was then dismissed with instructions to syringe out the cavity twice a day with the carbolic acid and iodin wash, as long as syringe could be used; afterward the medicament was to be used as a mouth wash. In five weeks new bone-tissue had filled the void, when no suspicion of the parts having been diseased remained, the outline being quite perfect, and all the tissues in a normal condition.

Case 2.—Mr. T., a lawyer, aged 40, of sanguino-bilious temperament, presented the following conditions: A fistulous opening situated to the right of the left inferior cuspid tooth, another to the left of the right inferior cuspid; both openings discharging pus copiously. This condition had continued for over a year, and Mr. T. failing to get any relief from many medications, he consulted a surgeon, who diagnosed his case as necrosis, caused, the surgeon said, by the toxic effect of mercury or syphilis. To either being the cause Mr. T. protested most vehemently. Mr. T. was informed that he would have to be operated upon. The first thing the surgeon suggested was to have all the incisor teeth extracted in the hope that nature would have a better chance to throw off the sequestrum. Mr. T. did not object to having the operation performed, but he did object most strenuously against having the teeth extracted, as he possessed a beautiful set, being perfect in form and arrangement. He consulted me to find out if I could in some way manage to save his teeth. I suggested the sulphuric acid treatment, which I thought would not only save his teeth, but save him from having to undergo any operation. After consulting with the surgeon it was agreed that I should take charge of the case, with the understanding that I was to consult with the surgeon as the case progressed, and not, under any circumstances, change treatment without his knowledge. The treatment in this case was the same as in case of number one. After the first day's treatment Mr. T. came to the office early in the morning with a very distressed countenance, and said he believed I had aggravated the case, because the flow of pus had been so great during the night that he could scarcely sleep. I assured him it was a good sign and that the remedy was doing its work well. The first week the teeth became movable and tender to the touch. A vulcanite splint plate was made to hold the teeth in place and to protect them from shock during the process of mastication. At the expiration of two weeks all discharge had ceased. Then an incision was made from the left fistulous opening to the right, the cavity packed with absorbent cotton saturated with the carbolic acid and iodin mixture (as in first case) and left to remain until next day. When pledget of cotton was removed the following conditions were presented: Line of necrosis found to have involved the facial surface ef the inferior maxilla, from cuspid to cuspid, slightly exposing the nerves at the apices of two central incisors. The septa were nearly destroyed, the teeth were only held in position by their attachment to the external or posterior plate of the alveolar process and gum tissue. Some instructions for injection and mouth wash were given as in former case. In six weeks Mr. T. was pronounced cured—teeth all living and firm, and the continuity of maxilla outline fully restored.

I take no credit for the successful treatment of these cases, but will give all honor and praise to Dr. Gross, of Philadelphia, who, I believe, was the first to suggest the sulphuric acid treatment for necrosis. Some one may ask the question: How does the sulphuric acid act? In reply I will say, that the bony structures of the human organism contains 33 per cent. organic matter, 4 per cent. water and 63 per cent. mineral matter, 60 per cent. of which is carbonate of lime. The action of the sulphuric acid is to seize chemically upon the carbonate of lime, break it down or dissolve it, throwing off carbon dioxid gas; and subsequently the animal tissues, etc., break up through a process of fatty degeneration and flow out in form of pus, etc. The acid in the strength prescribed has no effect upon healthy tissues, except to stimulate them. The advantage of this treatment in the oral cavity over that of operative is: The periosteum is in no way injured, thereby not causing a loss of the continuity of outline of any structures, nor the loss of teeth through the necessary extraction in removal of the sequestra.

Miss G., the first case described, told me not many weeks ago she had not fully appreciated what I had done for her until after she had made a visit to Germany, some three years ago. She informed me of a cousin living in Germany who had a similar case with her own, age the same, conditions about the same, but the treatment and and final result quite different. In her cousin's case the teeth involved had been extracted by a surgeon prior to the removal, by operation, of the sequestrum. During the operation the periosteum had been injured, causing a loss of a great part of the maxilla; in consequence her face was disfigured for life. Moreover, she had never been able to have the continuity of outline restored by any artificial means. Now, suppose the two cases I have cited had been treated by operation, in like manner, what would have been the consequence?

In conclusion, I would call the attention of the surgeon, no matter how prone he may be to operate in the oral cavity, not to do so before he has failed in the sulphuric acid treatment, or use more conservative surgery than is usually practiced in similar cases.

